# Parasite assemblages in volatile host stocks: inter- and intra-cohort variability restrict their value as biological tags for squid stock assessment

**DOI:** 10.1017/S0031182023001051

**Published:** 2023-11

**Authors:** María Paz Gutiérrez, Delfina Canel, Paola E. Braicovich, Ana L. Lanfranchi, Manuel M. Irigoitia, Marcela L. Ivanovic, Nicolás I. Prandoni, Beatriz Elena, Juan T. Timi

**Affiliations:** 1 Laboratorio de Ictioparasitología, Instituto de Investigaciones Marinas y Costeras (IIMyC), Facultad de Ciencias Exactas y Naturales, Universidad Nacional de Mar del Plata- Consejo Nacional de Investigaciones Científicas y Técnicas (CONICET), Buenos Aires, Argentina; 2 Instituto Nacional de Investigación y Desarrollo Pesquero (INIDEP), Buenos Aires, Argentina

**Keywords:** Argentina, biological tags, *Illex argentinus*, parasite assemblages, stocks

## Abstract

The Argentine shortfin squid, *Illex argentinus*, inhabits in the southwest Atlantic; it is a semelparous species which grows rapidly along its 1 year lifespan. The identification of its stocks is critical for sustainable fishery exploitation. Parasites have been used as biological indicators in a lower number of studies dealing with squids, therefore a validation of this methodology is necessary. The intra- and inter-cohort variability of parasite assemblages in the summer-spawning stock of *I. argentinus* was analysed to assess their value as indicators of stock structure. Four squid samples from the continental shelf of central Patagonia, corresponding to 3 consecutive cohorts, were examined for metazoan parasites. Results evidenced heterogeneity in terms of parasite assemblage composition and structure, dominated by short-lived gastrointestinal parasites, with a strong influence of host size, but no effect of squid sex. These changes are related to their recent habitats and diets, which change with ontogeny and migrations, clouding any interpretation of patterns when samples spatially or temporally separated are compared. Many squid species share these characteristics; therefore, it is recommended that the use of parasites as biological tags should be restricted to simultaneous sampling, while size or age must be considered for deriving proper conclusions.

## Graphical abstract

**Figure:**
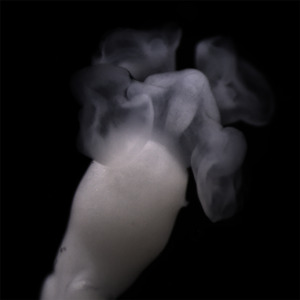

## Introduction

The Argentine shortfin squid *Illex argentinus* Castellanos, 1960 (Ommastrephidae) is a neritic-oceanic species, widely distributed along the outer shelf and slope of the south-western Atlantic Ocean, between 22°S and 54°S (Brunetti, 1988; Torres Alberto *et al*., 2020), however, most catches occur in the southern range of the species (35°S to 52°S) (Haimovici and Pérez, 1990; Haimovici *et al*., 1998, 2014). Two main currents (Malvinas and Brazil currents) dominate the regional oceanography, with their variability and interactions with other masses of water being the main determining factors of the Argentine shortfin squid distribution (Nigmatullin, 1989; Haimovici *et al*., 1998; Bazzino and Quiñones, 1999). Such temporal variations in environmental conditions are common processes in the region, resulting in changes in the abundance and availability of squid preys, which, in turn, explain the interannual fluctuations usually recorded in squid abundance (Bazzino and Quiñones, 1999). Furthermore, for *I. argentinus*, as for many other squid species, these strong interannual fluctuations in abundance are also a consequence of its semelparous life strategy and its latitudinal and bathymetric migrations, coupled with environmental influences on its recruitment (Dawe and Brodziak, 1998; Pierce *et al*., 2008; Torres Alberto *et al*., 2020).

*Illex argentinus* is a short-lived species, displaying a rapid growth rate and an annual life cycle, resulting in non-overlapping generations. It exhibits opportunistic trophic strategies, displaying a highly dynamic role in the trophic web, which can shift significantly between years and geographical areas due to variations in recruitment and in the abundance of interacting species within the food chain (Dawe and Brodziak, 1998).

**Figure 1.**
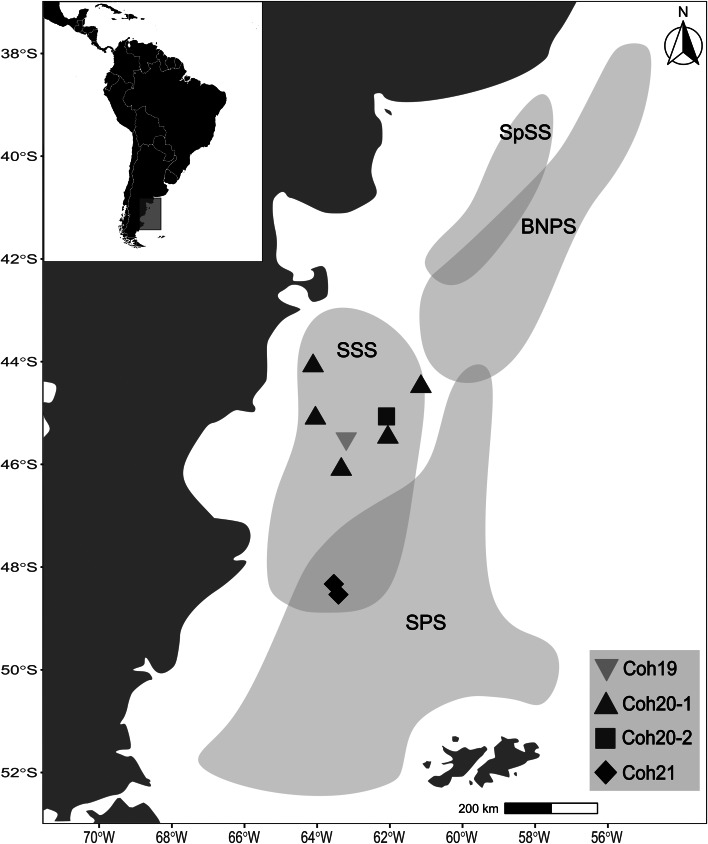
Study area showing the stocks distribution of *Illex argentinus*, spring-spawning stock (SpSS); bonaerensis-north patagonic stock (BNPS); summer-spawning stock (SSS); south patagonic stock (SPS). Coh19: cohort 2019; Coh20-1, Coh20-2: cohort 2020; Coh21: cohort 2021. Inverted triangles and squares represent a single station of Coh19 and Coh20-2 samples, respectively; whereas triangles and rhombuses belong to 5 and 2 stations of Coh20-1 and Coh21, respectively.

This is one of the most important commercial squid species for the Argentine fisheries, with total catches reaching 345 000 tons in 2020 (FAO, 2022). The assessment of their stocks is, therefore, critical for a sustainable fishing exploitation, but also for the maintenance of the ecological integrity of food webs given the relevance of the squids as both predator and prey (Vidal and Haimovici, 1999). The population structure of the southern range of *I. argentinus* is complex, with 4 stocks or subpopulations being differentiated according to the season and reproduction ground (Fig. 1): the spring-spawning stock (SpSS), the bonaerensis-north patagonic stock (BNPS), the summer-spawning stock (SSS) and the south patagonic stock (SPS) (Brunetti, 1988; Ivanovic *et al*., 2016; Arkhipkin *et al*., 2023). During the austral summer, from December to February, densest concentrations of *I. argentinus*, composed by juveniles and adults of 2 stocks (SPS and SSS, respectively), occur over the Patagonian continental shelf between 43°S and 55°S (Brunetti *et al*., 1998). The co-occurrence in this zone, at certain times of the year, takes place due to the migratory-reproductive annual cycle of SPS, determining a mixing zone between 47°S and 49°S, approximately (Avigliano *et al*., 2020). Stocks are distinguishable from each other by their size and their gonadal maturity. Indeed, reproductive squids, corresponding to SSS specimens, are found in the north region (44°–48°S), whereas pre-reproductive concentrations between 49°–52°S are represented by SPS individuals (Ivanovic *et al*., 2016). Furthermore, a comprehensive stock assessment is necessary because this species is exploited during its ontogenetic migrations, both within exclusive economic zones of different coastal states along South American coasts and in adjacent high seas (Arkhipkin *et al*., 2023). In the high seas, where squids harvest accounts for around 45% of the total catch, regulation and control of fisheries are non-existent, posing a serious risk of stock depletion becoming the resource highly vulnerable to overfishing during years of poor recruitment and low abundance (Arkhipkin *et al*., 2023), highlighting the need for their precise identification.

According to Timi and Buchmann (2023), the vast majority of studies on parasites as biological indicators to discriminate stocks deal with teleost fish as host, with a comparatively lower number of studies considering elasmobranchs and invertebrates. Despite the economic relevance of *I. argentinus* for regional fisheries and the knowledge about their parasites (Threlfall, 1970; Nigmatullin, 1989; Hochberg, 1990; Nigmatullin and Shukhgálter, 1990; Sardella *et al*., 1990; Vidal and Haimovici, 1999; González and Kroeck, 2000; Cipriani *et al*., 2019), a single study has used parasites as indicators for their stock assessment in San Matías Gulf, Argentina (González and Kroeck, 2000). However, results on parasite tags should be taken cautiously, and must consider the lifespan of squid parasites and its interaction with host characteristics, especially when infestations depend on host size or age (Lester and MacKenzie, 2009; Timi and Poulin, 2020). Indeed, the ecology and behaviour of cephalopods, together with life cycle, mantle size, deep range and vagility are important drivers of parasite diversity (González *et al*., 2003). Additionally, migratory species such as squids, which alternate between feeding and spawning habitats, can evidence ontogenetic changes in the structure of transient parasite assemblages, leading to misinterpretation of their stock structure.

In this paper, the variability of parasite loads due to hosts and environment is analysed for squids of the SSS stock because it is known that they perform only small-scale spatial migrations, restricted to the outer shelf at depths ranging between 50 and 200 m, not using high sea areas every year (Arkhipkin *et al*., 2023). This characteristic makes the SSS easier to analyse than the SPS stock, which undergoes extensive migrations and, as a result, introduces potential variability to parasite loads when moving through oceanographically distinct areas. The aim of this study is, therefore, to analyse the inter- and intra-cohort variability in the structure of parasite assemblages in the SSS of *I. argentinus* to assess their value as indicators of stock structure in further studies at broader scales.

## Materials and methods

### Squid and parasites sampling

A total of 318 specimens of *I. argentinus*, distributed in 4 samples, corresponding to 3 consecutive cohorts (Coh19, Coh20-1, Coh20-2, Coh21) of the SSS caught between 2020 and 2022, were examined for metazoan parasites (Table 1). Two of them, Coh20-1 and Coh21, were obtained from research cruises of the Instituto Nacional de Investigación y Desarrollo Pesquero (INIDEP), and those corresponding to Coh19 and Coh20-2 were obtained from commercial catches during summer, at intermediate waters of central Patagonia. Squids belonging to Coh20-1 and Coh21 included 5 and 2 stations, respectively, whereas Coh19 and Coh20-2 both included a single station (Fig. 1).

**Table 1.** Composition of 4 samples of *Illex argentinus* belonging to 3 consecutive cohorts of summer-spawning stock

Sample code	Cohort	Date of capture	Latitude/longitude	*n*	Mantle length ± s.d.
Coh19	2019	02/2020	45°30’S, 62°12’W	94	23.91 ± 2.90
Coh20-1	2020	12/2020	46°5’–44°4’S, 64°6’–62°3’W	160	20.39 ± 2.38
Coh20-2	2020	02/2021	45°S, 62°W	43	27.28 ± 1.86
Coh21	2021	02/2022	48°32’–48°19’S, 63°32’–63°25’W	21	24.14 ± 2.28

Squids were deep frozen in plastic bags at −18°C until the examination. After thawing, each squid was measured (dorsal mantle length [ML], cm) and cut along the ventral midline of the mantle. Furthermore, sex and gonadal maturity index according to an established scale of maturity (Brunetti *et al*., 1999) were determined.

The mantle, funnel, buccal cavity and viscera (oesophagus, stomach, digestive caecum, intestine, digestive gland, gills, heart, kidney and gonads) were examined and parasites were recovered and examined under a stereoscopic microscope. Some specimens were fixed on formalin 4% and ethanol 96% for morphological and molecular identification, respectively.

### Genetic identification of cestode larvae

Given the wide range of sizes and shapes of larval cestodes (plerocercoids) parasitizing *I. argentinus* (see Threlfall, 1970; Nigmatullin and Shukhgálter, 1990), and the presence of intermediate forms and sizes, it was necessary to assess how many taxa they represent. Therefore, a subsample of 8 plerocercoids from digestive tracts and 1 undeveloped larva found encysted in the stomach wall were collected for genetic analysis. DNA extraction was carried out using whole specimens with the DNeasy Blood and Tissue® Kit (Qiagen, Hilden, Germany) according to the manufacturer's instructions. A fragment (~1400 bp) of the lsrDNA gene (28S rDNA) spanning domains D1–D3 was amplified using the primer combinations ZX-1 (Waeschenbach *et al*., 2007)/1500R (Tkach *et al*., 2003) or LSU5 (Littlewood *et al*., 2000)/1200R (Lockyer *et al*., 2003). The polymerase chain reaction (PCR) reactions were carried out in a 25 *μ*L volume containing 0.5 *μ*L of each primer (10 mm), 3 *μ*L of MgCl2 25 mm (Promega, Wisconsin, USA), 5 *μ*L of 5 × buffer (Promega), 2 *μ*L of dNTPs 10 mm, 0.25 *μ*L of Go-Taq Polymerase (5 U *μ*L^−1^) (Promega), 5 *μ*L of total DNA (~30 ng *μ*L^−1^) and sterilized distilled water up to 25 *μ*L. PCR temperature conditions were the following: 94°C for 2 min (initial denaturation), followed by 40 cycles at 94°C for 30 s (denaturation), 56°C for 45 s (annealing), 72°C for 2 min (extension) and followed by post-amplification at 72°C for 7 min.

All amplified PCR products were verified in a 1.2% agarose gel. The successful PCR products were purified using QIAquick Gel Extraction Kit or QIAquick PCR purification Kit (Qiagen). Sequencing of both strands was carried out using ABI 3730XLs automated sequencer (Applied Biosystems, Macrogen, South Korea).

Sequences were edited and assembled in Proseq v.3.5 (Filatov, 2002) and deposited in the GenBank database. For identification, generated sequences were compared against the NCBI database using the BLAST algorithm (Sayers *et al*., 2022). Sequences are available from GenBank under accession numbers OR725126 to OR725133.

### Quantitative and similarity analysis of parasites

Each parasite was identified and counted and the prevalence and mean abundance for each species in each sample were calculated following Bush *et al*. (1997). The ML was compared across samples by a 1-way permutational multivariate analysis of the variance (PERMANOVA, Anderson *et al*., 2008) on Euclidean distances (1 × 4 factorial design, ‘sample’ as fixed factor), testing for main effects after 9999 permutations and subsequent *post-hoc* pairwise comparisons. Following Anderson *et al*. (2008), an unrestricted permutation of raw data was used as the method of permutation. Species richness (*S*) was calculated for each individual squid, and the mean values were compared across samples by a PERMANOVA analysis as in the case of ML, but applying a sequential sum of squares (type I SS) because samples were unbalanced (different numbers of squids examined by sample) and host size was included as a covariate (analysis of covariance [ANCOVA] model).

Multivariate analyses between samples were conducted using both Bray–Curtis index (based on abundances) and Jaccard index (based on presence/absence) for all possible pairs of hosts (infracommunities *sensu* Bush *et al*., 1997) from different samples. Due to the large differences in parasite loads across parasite species, data were square root-transformed prior to all analyses in order to downweigh the importance of most prevalent/abundant species, so that the less dominant species contribute in determining similarity among samples (Clarke and Gorley, 2015).

To evaluate if samples can be differentiated based on the abundance and composition of their parasite assemblages, non-metric multidimensional scaling (nMDS) of the both similarity matrices was performed between all infracommunities, and their centroid differences were visualized by means of bootstrap averaging based on repeated resampling (with replacement, 75 iterations) from the original dataset (Clarke and Gorley, 2015). Differences between infracommunities among samples were further examined using canonical analysis of principal coordinates (CAP) (Anderson and Willis, 2003; Anderson *et al*., 2008).

The structures of parasite infracommunities were compared between samples considering possible gender difference, introducing host ML as a covariable (ANCOVA model, 2 × 4 factorial design, samples and sex as fixed factors) and testing for main effects after 9999 permutations, using Bray–Curtis and Jaccard indices. Following Anderson *et al*. (2008), a permutation of residuals under a reduced model was used as the method of permutation. A sequential sum of squares (type I SS) was applied because the use of a covariate due to samples was unbalanced (different numbers of squids examined by sample). Where differences were detected by PERMANOVA, pairwise comparisons were used to determine which samples differed. All similarity and distance measures, as well as multivariate analyses were implemented in PRIMER V7 and PERMANOVA+ for PRIMER package (Anderson *et al*., 2008).

## Results

**Figure 2.**
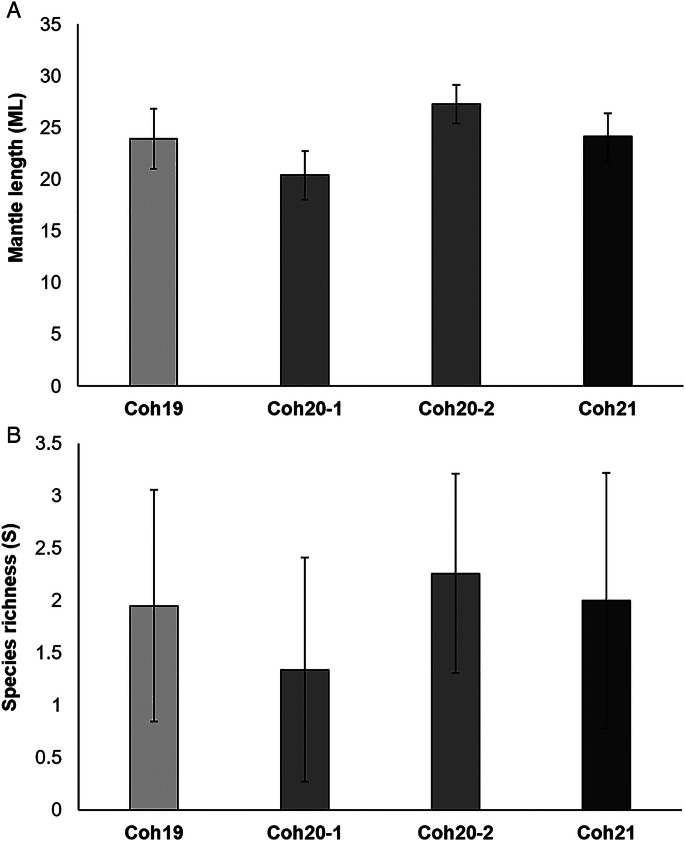
Averaged dorsal mantle length (A) and infracommunity species richness (B) of *Illex argentinus* in 4 samples of the summer-spawning stock. Cohorts are represented by a grey scale. Vertical bars representing standard deviations (as shown in Table 2). Coh19: cohort 2019; Coh20-1, Coh20-2: cohort 2020; Coh21: cohort 2021.

Squid ML was significantly different among samples (*F*_7,246_: 97.956; *P*_perm_ < 0.01) (Table 2, Fig. 2A), with pairwise comparisons showing significant differences (*P*_perm_ < 0.01) for most pairs of samples, except between Coh19 and Coh21 (*P*_perm_ > 0.05).

**Table 2.** PERMANOVA results of comparisons of mantle length, species richness, composition and structure of parasite communities of *Illex argentinus* across 4 samples corresponding to 3 cohorts of the summer-spawning stock

Response variability	Source	d.f.	SS	MS	*Pseudo F*	*P* (perm)
Univariate						
Mantle length	Cohort	3	1775.5	591.82	97.956	0.0001
(Euclidean distance)	Residual	267	1613.1	6.042		
	Total	270	3388.6			
Species richness	Mantle length	1	44.456	44.456	39.369	0.0001
(Euclidean distance)	Cohort	3	9.1348	3.0449	2.6965	0.0471
	Sex	1	1.0963	1.0963	0.97084	0.3312
	Mantle length × cohort	3	4.692	1.564	1.385	0.2476
	Mantle length × sex	1	6.0109e^−3^	6.0109e^−3^	5.3284e^−3^	0.9403
	Cohort × sex	3	7.1183	2.3728	2.1013	0.0916
	Residual	303	342.15	1.1292		
	Total	317	411.8			
Multivariate						
Infracommunity structure	Mantle length	1	70 597	70 597	25.774	0.0001
(Bray–Curtis similarity)	Cohort	3	39 459	13 153	4.8019	0.0001
	Sex	1	3952.8	3952.8	1.4431	0.2201
	Mantle length × cohort	3	11 802	3934.1	1.4363	0.137
	Mantle length × sex	1	5072.1	5072.1	1.8518	0.1094
	Cohort × sex	2	7619.7	3809.9	1.3909	0.1914
	Residual	259	7.09e^5^	2739.1		
	Total	270	8.48e^5^			
Infracommunity composition	Mantle length	1	65 643	65 643	22.773	0.0001
(Jaccard similarity)	Cohort	3	37 008	12 336	4.2797	0.0001
	Sex	1	2880.9	2880.9	0.99945	0.4104
	Mantle length × cohort	3	12 986	4328.5	1.5017	0.0987
	Mantle length × sex	1	4451.4	4451.4	1.5443	0.173
	Cohort × sex	2	6828.2	3414.1	1.1844	0.2963
	Residual	259	7.47e^5^	2882.5		
	Total	270	8.76e^5^			

*P* values obtained after 9999 permutations.

The molecular characterization allowed to identify 7 of the phyllobothriid plerocercoid sequences (1201 bp) as belonging to *Clistobothrium* n. sp. 1 (MT732134) and *Clistobothrium* sp. (KM272992 and MT732134) deposited in GenBank, with a percentage of identity of 99.67 (1 isolate) to 100% (6 isolates). Therefore, all morphological types of phyllobothriid plerocercoids were pooled for further analyses. The sequence of the undeveloped larval cestode (1430 bp) retrieved a percentage of identity of 96.65–96.79% with sequences of 3 unidentified species of *Grillotia* (Lacistorhynchidae) (MH688700, MH688704 and MH688707).

The whole sample of *I. argentinus* harboured 12 parasite taxa (Table 3). At infracommunity level, no differences in species richness were observed across samples (Table 2, Fig. 2B). A total of 85.22% squids were parasitized by at least 1 species, and 2187 individual parasites were recorded in the whole sample (Table 3). Only 8 of those taxa showed a prevalence >10% in at least 1 of the samples. Most parasites were larval forms, with the exception of the digeneans *Derogenes varicus* and *Elytrophalloides oatesi*, and the nematode *Hysterothylacium aduncum*, all found in the digestive tract. Larval cestodes dominated numerically the assemblages, representing 64.84% of individual parasites, being mainly represented by phyllobothridians and tetraphyllidians found in the digestive caecum, and less commonly in gills, stomach, intestine, funnel, buccal cavity and oesophagus.

**Table 3.** Prevalence (*P*), mean abundance (MA) with standard deviation (s.d.), site of infection and stage of development of parasites of *Illex argentinus* in 4 samples corresponding to 3 consecutive cohorts of the summer-spawning stock

Parasite	Site	Stage	Coh19		Coh20-1		Coh20-2		Coh21	
			*P*	MA ± s.d.	*P*	MA ± s.d.	*P*	MA ± s.d.	*P*	MA ± s.d.
**Nematoda**										
*Anisakis* sp.	Co	L3	46.81	1.05 ± 1.76	10.63	0.29 ± 1.79	81.40	2.23 ± 2.57	23.81	0.29 ± 0.56
*Hysterothylacium aduncum*	Di	L4/adult	19.15	0.27 ± 0.64	20.00	0.25 ± 0.55	16.30	0.16 ± 0.37	14.29	0.24 ± 0.62
*Hysterothylacium aduncum*	Co	L3	2.13	0.05 ± 0.37	3.75	0.04 ± 0.19	2.33	0.02 ± 0.15	–	–
Rhabditida fam. gen. sp. 1	Co	Larvae	41.49	0.86 ± 1.59	38.75	0.98 ± 1.70	39.53	0.67 ± 1.04	9.52	0.10 ± 0.30
Rhabditida fam. gen. sp. 2	Co	Larvae	10.64	0.11 ± 0.31	–	–	–	–	–	–
**Cestoda**										
*Clistobothrium* sp. 1^a^	Di	Plerocercoid	48.94	4.26 ± 17.72	30.00	1.48 ± 8.34	62.79	3.72 ± 14.07	80.95	7.24 ± 11.52
*Dinobothrium* sp. 1	Di	Plerocercoid	11.70	0.52 ± 2.35	2.50	1.21 ± 14.39	11.63	0.74 ± 2.73	57.14	9.19 ± 18.24
*Dinobothrium* sp. 2	Di	Plerocercoid	–	–	–	–	2.33	0.02 ± 0.15	–	–
Lacistorhynchidae gen. sp.^a^	St	Larvae	2.13	0.28 ± 1.94	10.63	0.43 ± 1.85	–	–	–	–
**Digenea**										
*Derogenes varicus*	Di	Adult	17.02	0.18 ± 0.41	17.50	0.21 ± 0.51	9.30	0.09 ± 0.29	14.29	0.24 ± 0.62
*Elytrophalloides oatesi*	Di	Adult	1.06	0.01 ± 0.10	–	–	–	–	–	–
**Isopoda**										
Gnathiidae gen. sp.	Ma	Praniza	–	–	0.63	0.01 ± 0.08	–	–	–	–

Di, digestive tract; Ma, mantle cavity; Co, coelomic membrane; St, stomach wall.

a Genetic identification.

**Figure 3.**
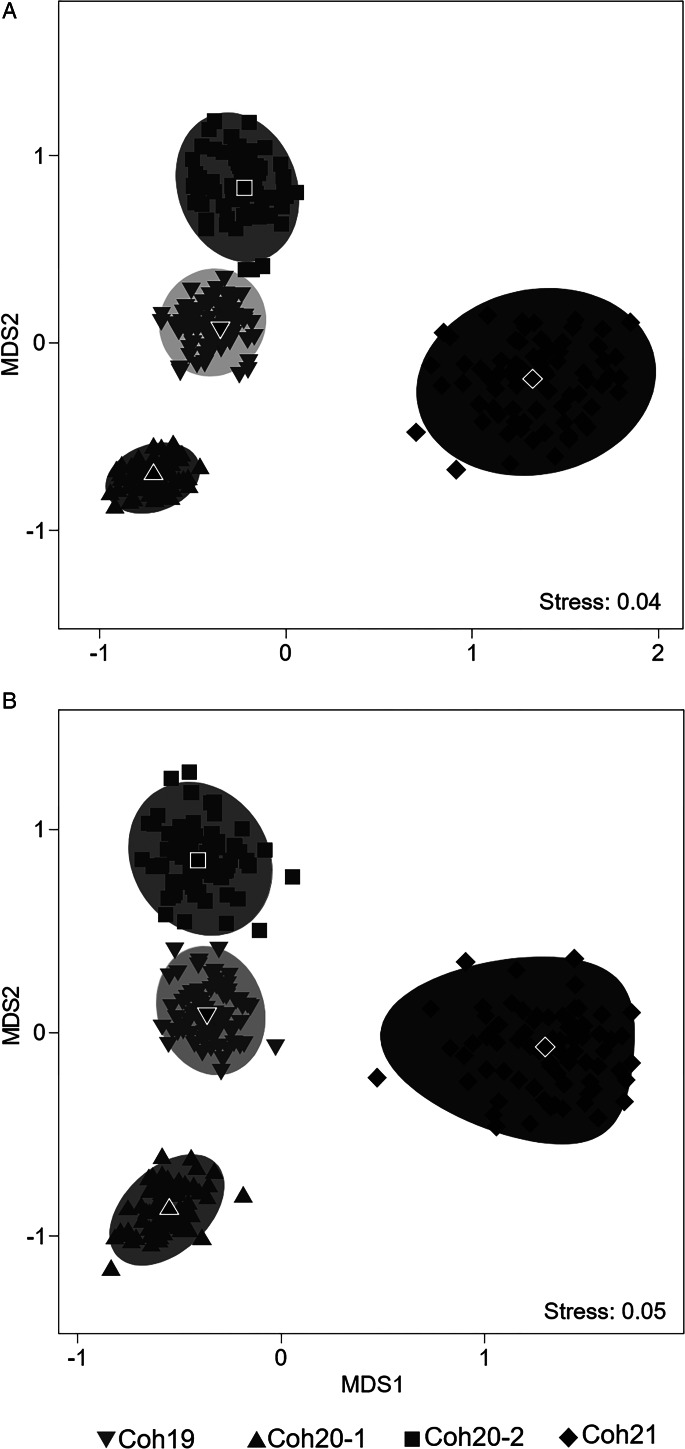
Non-metric multi-dimensional scaling plot (nMDS) of bootstrap averages (75 repetitions) of parasite infracommunities of *Illex argentinus* distributed within 4 samples at intermediate waters of central Patagonia based on Bray–Curtis and Jaccard similarity of square root-transformed data. Individual repetitions are based on random draw and replacement of samples from the original dataset. Black symbols represent the overall centroids across all repetitions. Grey areas represent 95% confidence regions. Coh19: cohort 2019; Coh20-1, Coh20-2: cohort 2020; Coh21: cohort 2021.

The bootstrap-average-based nMDS ordination of both Bray-Curtis (Fig. 3A) and Jaccard (Fig. 3B) was similar to each other, and showed an apparent pattern of separation between samples, with a low level of stress (0.04 and 0.05, respectively). Squids from Coh21 were clearly separated from the rest, especially along the first axis; parasite assemblages of Coh19 occupied an intermediate position between the 2 samples of Coh20, these 3 groups being mainly separated along the second axis.

The CAP analysis showed significant differences among samples (tr = 0.4725 and tr = 0.558 for Bray–Curtis and Jaccard, respectively; both *P* < 0.001). The selected orthonormal PCO axes (*m* = 5 and *m* = 6, respectively) described 92.5 and 99.15%, respectively, of the variation in the data ‘cloud’, although the percentage of correct allocations was relatively low (50.9 and 52.8%, respectively). Cross-validation results based on Bray–Curtis similarity (Table 4) showed that Coh21 had the highest percentage of correctly allocated squids, whereas most hosts from both samples of Coh20 were mostly allocated to their respective sample. Finally, Coh19 showed a very low proportion of correctly allocated squids, most of which were misclassified among Coh20-1 and Coh-20-2. When Jaccard index was considered (Table 4), a similar picture was observed, although several squids of Coh21 were misclassified in Coh19.

**Table 4.** Results of the cross-validation of principal co-ordinates analysis (CAP) based on Bray–Curtis and Jaccard similarity (leave-one-out allocation of individual squid to 1 of 4 samples)

Original sample	Coh19	Coh20-1	Coh20-2	Coh21	Total	% (group)
**Bray–Curtis**						
Coh19	*9*	30	36	11	86	10.47
Coh20-1	13	*80*	12	18	123	65.04
Coh20-2	2	5	*31*	4	42	73.81
Coh21	0	1	1	*18*	20	90
**Jaccard**						
Coh19	*10*	30	35	11	86	11.63
Coh20-1	17	*91*	11	4	123	73.98
Coh20-2	0	6	*31*	5	42	73.81
Coh21	6	1	2	*11*	20	55

Rows correspond to group memberships, including the percentage of correctly classified squid to their individual sample. Numbers in italics indicate number of squids in 4 samples of spawning-summer stock correctly allocated to their own locality.

The structure and composition of parasite infracommunities varied across samples and with ML but not with host sex, nevertheless no interaction between ML and samples was observed (Table 2). Pairwise comparisons after ‘correcting’ for the effect of squid length evidenced significant differences for both Bray–Curtis and Jaccard indices among most pairs of samples (*P*perm < 0.01), whereas squids from Coh19 were similar to those of both Coh20-1 and Coh20-2 (*P*perm > 0.05).

## Discussion

Previous records of parasites in *I. argentinus* from the south-western Atlantic Ocean are mostly descriptive, and include several species of cestodes, digeneans, nematodes, 1 copepod and 1 coccidia (Threlfall, 1970; Nigmatullin, 1989; Hochberg, 1990; Nigmatullin and Shukhgálter, 1990; Sardella *et al*., 1990; Vidal and Haimovici, 1999; González and Kroeck, 2000; Cipriani *et al*., 2019). To our knowledge, the present findings represent new host records for the digenean *E. oatesi*, the cestode Lacistorhynchidae gen. sp., larval nematodes of the Order Rhabditida and the gnathiid isopod.

In agreement with previous studies on parasite fauna of other cephalopod species (Pascual *et al*., 1995; Brickle *et al*., 2001; Tedesco *et al*., 2020), cestode plerocercoids were the most abundant group found in *I. argentinus*, which also showed a substantial morphological variability (see Threlfall, 1970; Nigmatullin and Shukhgálter, 1990; Sardella *et al*., 1990). Characterization of species *via* molecular analysis allowed to confirm that the larvae of *Phyllobothrium* sp., previously reported in *I. argentinus*, correspond to the phyllobothridean genus *Clistobothrium*, as was shown by Brickle *et al*. (2001) for cestodes from *Doryteuthis gahi* caught in southern areas. Similarly, the larval morphotype previously identified as *Pelichnibothrium speciosum* (Threlfall, 1970; Nigmatullin and Shukhgálter, 1990; Sardella *et al*., 1990) was genetically identified as *Clitobothrium*. The incipient development of the larvae that was found to be encysted did not allow their identification as a trypanorhynch cestode due to the lack of structures such as tentacles or bothridia. The absence of these structures has been observed in early stages of the larval development in some trypanorhynchs, such as those of the genus *Grillotia* (Schramm, 1991). Indeed, the highest percentages of identity were concordant with sequences of 3 unidentified species of *Grillotia* found in *Amblyraja radiata*, *Bathyraja magellanica* and *B. brachyurops*, respectively, the last 2 from South Atlantic Ocean (Beer *et al*., 2019).

Many studies on parasites of cephalopods are aged and reliant upon morphological features. Only recently, the implementation of molecular techniques began to more accurately elucidate the taxonomic status of cestode species parasitizing squids (Brickle *et al*., 2001; Caira *et al*., 2020; Guardone *et al*., 2020), providing evidence of a potential overestimation of cestode species in *I. argentinus* and allowing proper comparisons for squid stock assessment.

Host size is an important feature that affects parasite diversity by influencing the rates of colonization by new parasites (Luque and Poulin, 2004). Furthermore, host size is a useful surrogate of trophic level, determining patterns of predation and vulnerability to mortality (Jennings *et al*., 2001). Consequently, the characterization of the relationship between fish size and diet and trophic level is essential for assessing how much these interactions affect the structure of parasite assemblages (Timi *et al*., 2010, 2011). In the present study, whereas the increase of ML in Coh20-2 regarding Coh20-1 is an expected result, since the 2 months separating samples represent a considerable proportion of the annual squid lifespan, the observed variability in ML across years, even when squids were caught in the same month, can be attributed to the fact that growth and recruitment of cephalopods are highly influenced by environmental conditions. This results in wide inter-annual fluctuations of these processes, and even a sub-structure of microcohorts (intra-annual) is frequently present (Boyle and Rodhouse, 2005). Regarding parasites, the mean infracommunity species richness was uniform across samples. Such homogeneity responds to the small number of species found in the whole sample and the recurrence in the dominance by the same group of species, independently of their variability in abundance across samples. In the region, the diet of squids has evidenced a very low diversity and the dominance of a single type of prey in the majority of stomachs so far examined (Ivanovic and Brunetti, 1994; Ivanovic, 2010; Prandoni, 2022). Considering that most parasites of *I. argentinus* are acquired through the consumption of parasitized preys, the low diversity in its food items explain the rather homogeneous species richness recorded.

Beyond the similar values of species richness, all multivariate analyses evidenced a high level of structural and compositional heterogeneity across samples, largely driven by differences in squid size, but not by sex. Given that most parasites are transmitted trophically to squids, changes in parasitism are surely determined by host diet and its ontogenetic and seasonal changes. Transient parasites, namely those living in the gut lumen of hosts, such as most helminths found in *I. argentinus*, can persist in the host for a few weeks (Lester *et al*., 1985; Lester, 1990) representing mostly the food items consumed in recent times, whereas long-lived parasites, such as larvae of *Anisakis*, tend to accumulate as the host ages (Braicovich *et al*., 2016). This cumulative pattern is readily evident when comparing the increase of prevalence in squids from the same cohort, caught 2 months later.

The dominance of short-lived helminths makes the parasite assemblages of the Argentine shortfin squid highly susceptible to short-term environmental fluctuations, either directly or indirectly through their effect on zooplankton and other intermediate hosts. Consequently, depending on recent diets, their composition and structure can vary accordingly, driving unpredictable temporal patterns. Indeed, the observed interannual changes were not uniform, with parasite communities of Coh19 and Coh20 being more similar to each other than to Coh21. Similar heterogeneities were observed even for members of the same cohort. Of course, these differences are influenced by the differences in squid size and the locality of capture, but even after correcting for host size, most differences remained. On the other hand, squid sex had no effect on parasite loads. This is a consequence of the numerical preponderance of trophically transmitted parasites in a host that does not exhibit gender differences in diet composition or relative abundance (Prandoni, 2022), nor in somatic growth rates prior to sexual maturity (Lin *et al*., 2015). Likewise, no differences between sexes were observed for the parasites of *Illex coindetii* in Galician waters, Spain (Pascual *et al*., 1995).

In addition to the features of the parasite taxa, as well as of the host diet and its variations, other host and environmental characteristics play crucial roles in determining the observed patterns of parasite variability. Indeed, *I. argentinus*, like many other cephalopods, exhibits high metabolic rates and rapid growth, whose high variability is environmentally driven, resulting in interannual variations in stock abundance and distribution (Arkhipkin *et al*., 2021). Furthermore, its life cycle is highly variable both spatially and temporally due to their latitudinal migrations and bathymetric distribution, the latter also changing throughout its ontogeny (Ivanovic *et al*., 2016), all features that make their trophic interaction quite dynamic and scale-dependent.

In relation to the environment, spawning of *I. argentinus* is associated with marine fronts, whose characteristics change seasonally and their geographical locations vary according to the dynamics of marine currents in semi-annual, annual and biannual cyclic periods (Acha *et al*., 2004; Sacau *et al*., 2005). Indeed, studies modelling squid abundance in this ecosystem have retained sea surface temperature, latitude, longitude, month, average fishing depth and year as main predictors (Sacau *et al*., 2005), evidencing the complexity of the system and justifying its variability. Disparate environmental conditions, when experienced by early stages of *I. argentinus*, can also affect the success of recruitment (Torres Alberto *et al*., 2020) and growth rates (Arkhipkin, 1990; Haimovici *et al*., 1998), leading to interannual differences in growth either intracohort and between winter stocks (Arkhipkin and Scherbich, 1991; Haimovici *et al*., 1998).

All these sources of variability related to parasites, hosts and environment are expected to be reflected on parasite assemblages of *I. argentinus*, requiring consequently a careful selection of those parasite tags to be used or, at least, to make cautious interpretations of the observed patterns. In view of this generalized variability, stock differences can be overestimated, undermining its value as management tools for sustainability of the resource. For such purposes, and to promote a rational exploitation of squids, differences between stocks of *I. argentinus* using parasite tags should be based on specimens of equivalent size or age, and caught simultaneously at the putative stock units under study. Any departure of these conditions poses a serious risk of misinterpreting natural variability due to the stock of origin. This seems to be the case of the previous study on parasite tags for *I. argentinus* (González and Kroeck, 2000), where differences in parasite fauna were attributed to variations between stocks arriving with months of differences to a north patagonian gulf and showing notable differences in ML. Even when the stock identification based on squid size and maturity could be correct, the variability in parasite loads may respond to different causes, such as to squid size and not to the stock of origin. Indeed, higher species richness and abundances were observed in a sample composed by squids ranging between 23 and 38 cm ML, in relation to the second sample whose members measured between 14 and 22 cm ML. Although a gradual increase of parasitism is acknowledged (González and Kroeck, 2000), host size seems to be overlooked as a relevant determinant of these changes. Furthermore, the authors argue that these stocks are biotopically isolated from those inhabiting neighbouring areas when compared with previous studies carried out a decade earlier.

The biological and ecological host features regulating the prevalence and abundance in parasite assemblages, mostly composed of transient parasites, are shared by many squid species. Research on parasites as tags has been misleading as an *ad hoc* tool in elucidating the discreteness of unit stocks (Pascual and Hochberg, 1996). Few previous works using parasites as indicators of stock structure for squids have been carried out worldwide, being not free of these flaws, or having concluded that parasites are of little value as biological tags. For example, Dawe *et al*. (1982) analysed the parasites of the short-finned squid *Illex illecebrosus* of several sizes caught at several localities, seasons and years in the northwest Atlantic. The authors concluded that, because of the broad geographic distribution of parasites, their condition of generalists and the lack of taxonomic resolution, parasites were useless as indicators. On the other hand, Bower and Margolis (1991) proposed parasites of the flying squid, *Ommastrephes bartrami*, as potential tags for discriminating stocks between north-western and north-eastern Pacific waters based on differences, after comparing their results with previous studies carried out years earlier and not considering squid size or age as relevant variables. Pascual *et al*. (1995) conducted a survey of parasites of short-finned squid *I. coindetii* taken from 2 locations off the north-western Iberian Peninsula. Despite finding geographic differences, parasite infections showed close correlations with host life-cycle, with parasite loads increasing often with host size and maturity, which were considered for interpreting the observed patterns. In a multidisciplinary study of *Nototodarus sloani* from New Zealand waters, carried out by Smith *et al*. (1981), parasitological evidence, after taking account of host age (or length) and month of capture, supported the results of genetic and morphological study resulted in the identification of 2 congeneric host species.

Owing to the short life-cycles and variable growth rates of most cephalopods, their stocks may be highly volatile, due to their susceptibility to recruitment and overfishing (Pierce and Guerra, 1994). Therefore, if parasites are selected as indicators for stock assessment, it is crucial to carefully consider all the previously discussed sources of variability in order to obtain reliable results. Otherwise, differences between host populational units could be overestimated or, at least, derived from parasite tags varying for reasons other than those driving to actual dissimilarities between host stocks.

## Data Availability

Datasets are available from the corresponding author upon reasonable request. Nucleotide sequences of the 28S rDNA of Clistobothrium sp. (OR725126 to OR725132) and Lacistorhynchidae gen. sp. (OR725133) were deposited in GenBank database (http://www.ncbi.nlm.nih.gov).
